# Vinpocetine alleviate cerebral ischemia/reperfusion injury by down-regulating TLR4/MyD88/NF-κB signaling

**DOI:** 10.18632/oncotarget.20699

**Published:** 2017-09-07

**Authors:** Li-Rong Wu, Liang Liu, Xiao-Yi Xiong, Qin Zhang, Fa-Xiang Wang, Chang-Xiong Gong, Qi Zhong, Yuan-Rui Yang, Zhao-You Meng, Qing-Wu Yang

**Affiliations:** ^1^ Department of Neurology, Xinqiao Hospital, The Third Military Medical University, Shapingba, Chongqing, China

**Keywords:** vinpocetine, cerebral ischemia/reperfusion, toll-like receptor 4, ischemic stroke, middle cerebral artery occlusion

## Abstract

Inflammatory responses play crucial roles in cerebral ischemia/reperfusion injury. Toll-like receptor 4 (TLR4) is an important mediator of the neuroinflammatory response to cerebral ischemia/reperfusion injury. Vinpocetine is a derivative of the alkaloid vincamine and exerts an anti-inflammatory effect by inhibiting NF-κB activation. However, the effects of vinpocetine on pathways upstream of NF-κB signaling, such as TLR4, have not been fully elucidated. Here, we used mouse middle cerebral artery occlusion (MCAO) and cell-based oxygen-glucose deprivation (OGD) models to evaluate the therapeutic effects and mechanisms of vinpocetine treatment. The vinpocetine treatment significantly reduced mice cerebral infarct volumes and neurological scores. Moreover, the numbers of TUNEL+ and Fluoro-Jade B+ cells were significantly decreased in the ischemic brain tissues after vinpocetine treatment. In the OGD model, the vinpocetine treatment also increased the viability of cultured cortical neurons. Interestingly, vinpocetine exerted a neuroprotective effect on the mouse MCAO model and cell-based OGD model by inhibiting TLR4-mediated inflammatory responses and decreasing proinflammatory cytokine release through the MyD88-dependent signaling pathway, independent of TRIF signaling pathway. In conclusion, vinpocetine exerts anti-inflammatory effects to ameliorate cerebral ischemia/reperfusion injury in vitro and in vivo. Vinpocetine may inhibit inflammatory responses through the TLR4/MyD88/NF-κB signaling pathway, independent of TRIF-mediated inflammatory responses. Thus, vinpocetine may be an attractive therapeutic candidate for the treatment of ischemic cerebral injury or other inflammatory diseases.

## INTRODUCTION

Stroke is associated with high rates of mortality and morbidity, leading to a substantial economic and societal burden.[1, 2] According to an epidemiological study, approximately eighty percent of stroke cases are caused by cerebral ischemia.[3] Currently, thrombolytic agents are the only clinical effective treatment for ischemic stroke, but the narrow therapeutic window and other safety concerns have limited their application.[4] The pathophysiological mechanisms of cerebral ischemic injury are complex and have not been fully elucidated. Inflammation has recently been shown to play an essential role in secondary brain injury following cerebral ischemia.[5–7]

Toll-like receptors (TLRs) are type I transmembrane proteins that play critical roles in the induction of immune and inflammatory responses by recognizing pattern-associated molecular patterns (PAMPs) and damage-associated molecular patterns (DAMPs).[8–10] Importantly, Toll like receptor 4 (TLR4) is the best studied TLR and plays important roles in diseases of the central nervous system.[11, 12] To our knowledge, two pathways are involved in TLR4 signal transduction to induce the release of proinflammatory cytokines, including the MyD88 (myeloid differentiation factor 88)-dependent pathway and TRIF (Toll/IL-1R domain-containing adaptor protein inducing interferon-beta)-dependent pathway.[13] In addition, the TLR4/MyD88/NF-kB signaling pathway mediates the release of proinflammatory cytokines and induces neuronal degeneration and apoptosis.[6, 12] Indeed, as shown in previous studies by our and other groups, inhibition of the TLR4 signaling pathway in mice protects against ischemic stroke.[14, 15]

Vinpocetine is an alkaloid extracted from periwinkle plant, which is produced by slightly altering the vincamine molecule.[16, 17] In the clinic, vinpocetine has been widely used to treat cognitive impairments and prevent cerebrovascular disease for many years.[18–22] In addition, the safety and efficacy of vinpocetine have been confirmed in many clinical trials.[23] Vinpocetine has been shown to act as a cerebral vasodilator to regulate brain blood flow.[24] However, vinpocetine has recently been shown to exert an anti-inflammatory effect. For example, according to the study by Jeon et al., vinpocetine inhibits TNF-α-induced NF-κB activation and the subsequent expression of proinflammatory mediators in multiple cell types, including vascular smooth muscle cells, endothelial cells, macrophages, and epithelial cells.[25] Consistent with these results, Ruiz-Miyazawa et al. also observed that vinpocetine inhibited the release of inflammatory factors, including TNF-α, IL-1β and IL-33, in a lipopolysaccharide-induced model.[26] Based on these studies, vinpocetine exerts its anti-inflammatory effect by inhibiting NF-κB activation. However, the effects of vinpocetine on the upstream pathways of NF-κB signaling, such as TLR4, have not been fully elaborated. Thus, we hypothesized that vinpocetine may alleviate cerebral ischemia and reperfusion injury by down-regulating TLR4/MyD88/NF-κB signaling.

In this study, we found vinpocetine exerts a protective role on a cerebral ischemia and reperfusion model by inhibiting TLR4/MyD88/NF-κB signaling, but not the TLR4/TRIF/NF-κB signaling pathway, in vitro and in vivo. Thus, our studies are the first to show that vinpocetine also exerts an anti-inflammatory effect by inhibiting the TLR4/MyD88/NF-κB signaling pathway in a cerebral ischemia and reperfusion model.

## RESULTS

### Vinpocetine reduced cerebral ischemia reperfusion injury

**Figure 1:**
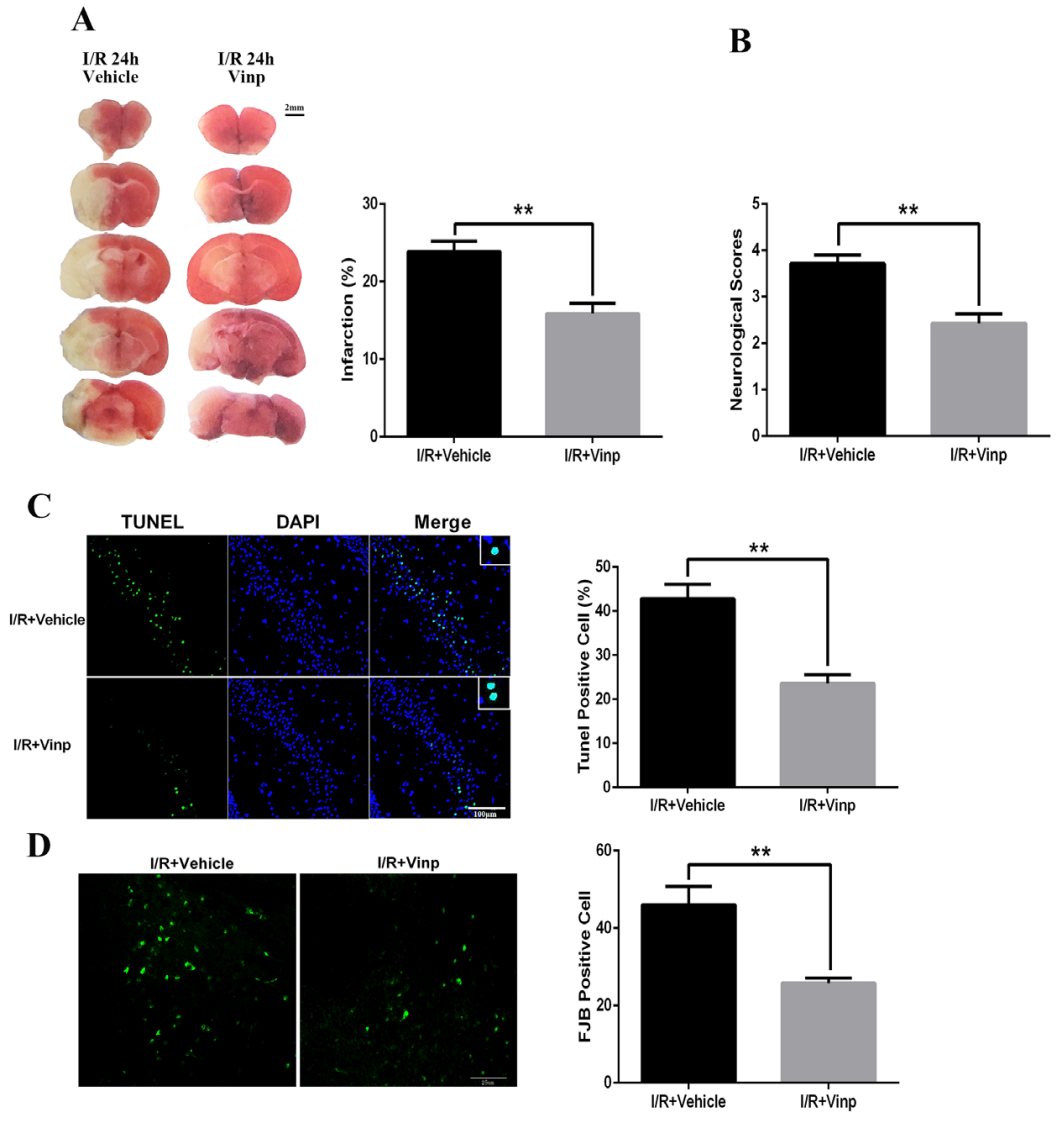
Vinpocetine reduced cerebral ischemia reperfusion injury After cerebral ischemia/reperfusion injury, Vinpocetine (10 mg/kg) or vehicle was i.p. injected into mice. **A.** TTC staining and quantification of the infarct volumes (*n* = 5). **B.** The vinpocetine treatment significantly decreased the neurological scores (*n* = 7) and numbers of TUNEL-positive **C.** and FJB-positive cells **D.** (*n* = 5). Data are presented as means±SEM (**p < 0.01).

Mice were i.p. injected with vinpocetine (10 mg/kg) after cerebral ischemia reperfusion injury to evaluate the protective effects of vinpocetine on cerebral I/R injury. Twenty-four hours after reperfusion, the brain infarction volume was analyzed using TTC staining. The vinpocetine treatment significantly reduced the cerebral infarct volume compared with the I/R group (Figure 1A). Consistent with the reduction in the brain infarction volume, we also observed a decrease in the neurological scores in the I/R+Vinp group (Figure 1B). TUNEL staining is a molecular biological- histochemical system that sensitively and specifically labels fragmented DNA. Twenty-four hours after reperfusion, brain sections were stained with TUNEL and Fluoro-Jade B (FJB), markers of apoptotic and degenerating cells. The vinpocetine treatment significantly reduced the numbers of TUNEL-positive (Figure 1C) and FJB-positive cells (Figure 1D).

### Vinpocetine increased neuronal viability and the LDH Levels and reduced neuronal apoptosis in the OGD model

**Figure 2:**
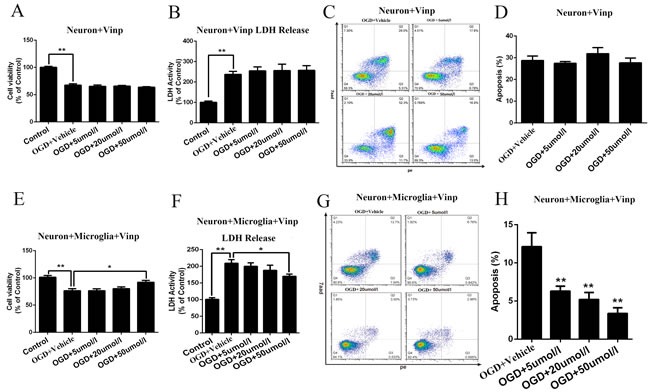
Vinpocetine increased the viability and LDH levels in primary cortical neurons and reduced neuronal apoptosis in the OGD model Primary cortical neurons and microglial cells were used for the OGD model, and the viability of primary cortical neurons and microglial cells was significantly decreased in response to OGD. However, we did not observe any significant effects of the vinpocetine treatment on primary cortical neuron viability **A.**, LDH release **B.** or cell apoptosis **C.**, **D.** after the OGD treatment. After microglial cells were subjected to OGD, their supernatants were added to primary cortical neurons and the effects of different concentrations (5, 20, and 50 μmol/L) of vinpocetine on primary cortical neurons viability **E.**, LDH release **F.** and apoptosis **G.**, **H.** were examined. Data are presented as means±SEM (*p < 0.05, **p < 0.01).

Primary cortical neurons and microglial cells were used for the OGD model. Moreover, different concentrations (5, 20, or 50 μmol/L) of vinpocetine was added to the culture medium prior to the induction of OGD in primary cortical neurons. Then, cell viability, LDH release and cell apoptosis were detected. However, we did not observe any significant effects of the vinpocetine treatment on primary cortical neuron viability (Figure 2A), LDH release (Figure 2B) and cell apoptosis (Figure 2C, Figure 2D) after OGD.

Different concentrations (5, 20, or 50 μmol/L) of vinpocetine were added prior to the induction of OGD in microglial cells to evaluate the effects of the vinpocetine treatment on microglial cells and the possible influence of microglial cells on neurons. Then, the microglial cell supernatant was collected and cultured with cortical neurons for 3 h. Interestingly, primary cortical neuron apoptosis was attenuated in the presence of the microglial cell supernatant. In addition, primary cortical neuron viability was significantly decreased and LDH release was increased after the microglial cell supernatant was added. However, these effects were blocked by treatment with a high concentration of vinpocetine (50 μmol/L) after microglial cells were subjected to OGD (Figure 2E-2H).

### Vinpocetine inhibited the activation of the TLR4/MyD88/NF-κB pathway in mice with cerebral ischemia-reperfusion injuries and the cell-based OGD model

**Figure 3:**
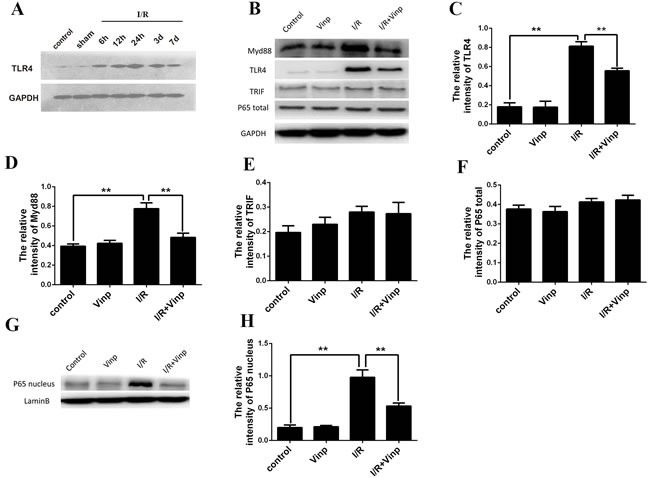
Vinpocetine inhibited the activation of the TLR4/MyD88 /NF-κB pathway in mice with cerebral ischemia reperfusion injury **A.** After cerebral ischemia/reperfusion injury, TLR4 expression was increased significantly in the infarcted area 24 h after the induction of ischemia and reperfusion. **B.** The levels of TLR4, MyD88, TRIF, and total and nuclear NF-kB p65 proteins were examined by Western blotting. The vinpocetine treatment inhibited the increase in TLR4 **C.** and MyD88 **D.** expression, but not TRIF **E.** (*n* = 5). Total NF-κB p65 **F.** and nuclear NF-κB p65 **G.**, **H.** expression are shown. Data are presented as means±SEM (**p < 0.01).

According to the results of a previous study, the TLR4 pathway plays a crucial role in cerebral ischemia reperfusion injury.[27] TLR4 expression was significantly increased in the infarcted area 24 h after the induction of ischemia and reperfusion (Figure 3A). To our knowledge, MyD88- and TRIF-dependent pathway are two common pathways involved in TLR4 signal transduction that induce the release of proinflammatory cytokines. Interesting, the MyD88 and TRIF pathways were activated in response to ischemia-reperfusion injury. However, the vinpocetine treatment inhibited the increase in TLR4 (Figure 3B and 3C) and MyD88 (Figure 3B and 3D) expression, but not TRIF (Figure 3B and 3E). Consistent with the results of a previous study using a TNF-α-induced NF-κB activation model,[25] vinpocetine also inhibited NF-κB activation in the MCAO model (Figure 3B, 3F, 3G and 3H).

### In the cell-based OGD model, vinpocetine also could inhibit the activation of the TLR4/MyD88/NF-κB pathway

**Figure 4:**
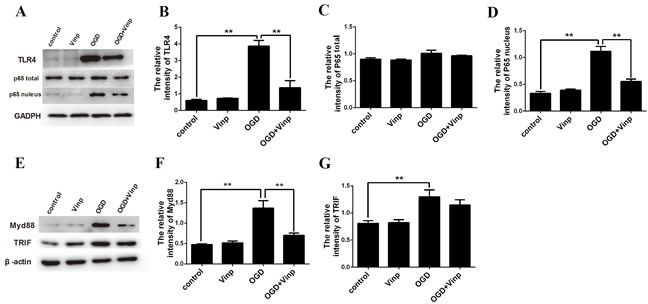
Vinpocetine inhibited the activation of the TLR4/MyD88 /NF-κB pathway in the microglia OGD model **A.** Effects of the 50 μmol/L vinpocetine treatment on the levels of the TLR4, total and nuclear NF-κB p65 proteins were examined by Western blotting. The vinpocetine treatment inhibited the increase in TLR4 **B.** Total NF-κB p65 **C.** and nuclear NF-κB p65 **D.** expression are shown. **E.**-**G.** The vinpocetine treatment inhibited the increase in MyD88 but not TRIF. Data are presented as means±SEM (**p < 0.01).

Similar results were obtained in the microglia OGD model. The vinpocetine treatment decreased the levels of activated TLR4 (Figure 4A and 4B) and NF-κB activation (Figure 4A, 4C and 4D) in the microglia OGD model. Moreover, the MyD-88 and TRIF signaling pathway were also activated in microglial cells in response to OGD. However, vinpocetine treatment only alleviating the MyD-88 signaling pathway (Figure 4E-4G).

### Vinpocetine attenuated inflammatory cytokine release in mice with cerebral ischemia-reperfusion injuries and the cell-based OGD model

**Figure 5:**
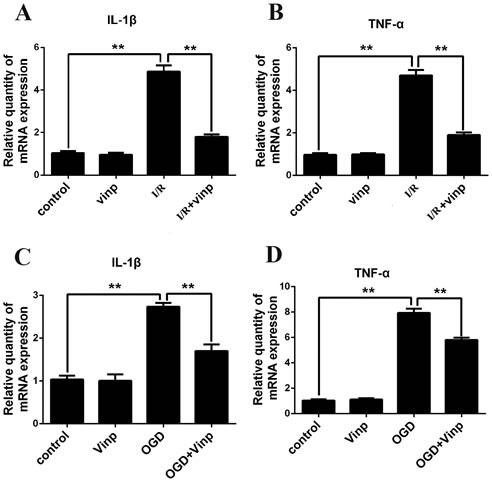
Vinpocetine attenuated inflammatory cytokine release in mice with cerebral ischemia-reperfusion injuries and the cell-based OGD model Effects of vinpocetine on the secretion of IL-1β **A.** and TNF-α **B.** in the mouse cerebral ischemia-reperfusion injury model are shown. Effects of vinpocetine on the secretion of IL-1β **C.** and TNF-α **D.** in the microglia OGD model are shown. Data are presented as means±SEM (**p < 0.01).

Inflammatory cytokine release was examined by RT-PCR to identify changes in the factors downstream of the TLR4/MyD88/NF-κB pathway. Twenty-four hours after the induction of ischemia and reperfusion, the levels of the IL-1β and TNF-α mRNAs were significantly increased in vivo. Indeed, the vinpocetine treatment alleviated those effects (Figure 5A and 5B). Similar results were also obtained in the in vitro study. In the OGD model, the microglia-mediated release of the inflammatory cytokines TNF-α and IL-1β was significantly increased. The vinpocetine treatment also attenuated the release of the inflammatory cytokines IL-1β and TNF-α in the microglia OGD model (Figure 5C and 5D).

## DISCUSSION

In this study, we identified the neuroprotective effects of vinpocetine on cerebral I/R injury in vitro and in vivo. First, the vinpocetine treatment attenuated TLR4 signaling via the MyD88-dependent pathway, but not the TRIF-dependent pathway, to protect against cerebral I/R injury in vitro and in vivo. As shown in a previous study, vinpocetine inhibits TNF-α-induced NF-κB activation by directly targeting IκB kinase (IKK) in several cell types.[25] We showed here, that vinpocetine protects against cerebral I/R injury by inhibiting NF-κB activation.

Vinpocetine is a derivative of the alkaloid vincamine and has been used to treat cerebrovascular disorders and cognitive impairment in the clinic for many years.[28] As shown in a rat MCAO model, vinpocetine is a unique anti-inflammatory agent that increases the resistance of the brain to hypoxia and ischemic injury and protects against cerebral I/R injury by inhibiting the functional expression of NF-κB.[19, 29] Vinpocetine exerted an anti-inflammatory effect on cerebral I/R injury in both a mouse MCAO model and a microglia OGD model in this study. Proinflammatory cytokines, such as TNF-α and IL-1β, produced by microglia are involved in ischemic neuronal injury and aggravate neurologic deficits.[30] The vinpocetine treatment significantly decreased TNF-α and IL-1β release in a mouse MCAO model and a microglia OGD model. Thus, vinpocetine attenuated ischemic neuronal injury and aggravated neurological deficits by inhibiting NF-κB activation and proinflammatory cytokine release.

The TLR4-mediated inflammatory response plays a crucial role in cerebral ischemia-reperfusion injury. TAK242, a TLR4 antagonist, exerts a neuroprotective effect after cerebral ischemia-reperfusion injury.[27] As shown in our study, vinpocetine also inhibited TLR4 expression to attenuate the excessive and uncontrolled inflammatory response after cerebral ischemia-reperfusion injury. To our knowledge, TLR4 is mainly located in microglial cells.[31, 32] Microglial cells act as a “double-edged sword” within the CNS by mediating the immune response and maintaining homeostasis.[33] Ischemic stroke stimulates endogenous inhibitory signaling and triggers microglial activation. Importantly, TLR4 regulates microglial activation and production of inflammatory mediators.[32] In the present study, 50 μmol/L vinpocetine inhibited TLR4 expression in microglial cells after OGD. In contrast to the results of a previous study, vinpocetine inhibited TNF-α-induced NF-κB activation by directly targeting IKK.[25] This discrepancy may be attributed to the various negative regulatory mechanisms involved in regulating TLR-mediated immune responses.[34] Based on these data, the inhibitory effect of vinpocetine on the inflammatory response induced by ischemia-reperfusion injury may also be involved in TLR4 signaling, thereby revealing a previously undescribed action of vinpocetine.

Furthermore, within the TLR4 signaling pathway, the MyD88-dependent and TRIF-dependent signaling pathways are two important activators of NF-κB and the subsequent regulatory effects of NF-κB signaling.[35] As reported in a previous study, a Dioscin treatment ameliorates cerebral I/R injury by down-regulating the TLR4/MyD88 signaling pathway.[36] Interestingly, in our present study, the level of MyD88 expression was reduced by vinpocetine treatment after cerebral ischemia/reperfusion injury or OGD. However, vinpocetine did not reverse the increase in TRIF expression induced by cerebral ischemia/reperfusion injury or OGD. Based on these data, vinpocetine inhibits NF-κB activation through the MyD88-dependent signaling pathway, but not the TRIF-dependent signaling pathway, in response to cerebral ischemia/reperfusion injury or OGD. Besides, consisted to previous studies findings,[26, 37] we also found vinpocetine inhibit NF-κB activation and downstream cytokines, including IL-1β and TNF-α.

In conclusion, vinpocetine acts as an anti-inflammatory agent by ameliorating cerebral ischemia/reperfusion injury in vitro and in vivo. Vinpocetine inhibits inflammatory responses through the TLR4/MyD88/NF-κB signaling pathway that are independent of TRIF-mediated inflammatory responses. Thus, vinpocetine may be an attractive therapeutic candidate for the treatment of cerebral ischemic injuries or other inflammatory diseases.

## MATERIALS AND METHODS

### Animals

Age- and weight-matched male C57BL/6 mice (8–12 wks old, 20–23 g) were obtained from the Laboratory Animal Center of The Third Military Medical University (Chongqing, China). All animals were provided free access to food and water in a clean environment. Experiments were conducted in accordance with animal care guidelines of the Animal Ethics Committee of TMMU (SYXK-PLA-2015034) and ARRIVE (Animal Research: Reporting In Vivo Experiments).

### MCAO model and vinpocetine treatment

The middle cerebral artery occlusion (MCAO) model was employed as described in our previous study.[14] Briefly, the mice were first anesthetized with 5.0% isoflurane. Then, an incision was made in the skin and the right common carotid artery (CCA), external carotid artery (ECA), and internal carotid artery (ICA) were carefully exposed. Microvascular aneurysm clips were applied to the right CCA and the ICA. A coated 6-0 filament (Doccol, Redlands, CA) was introduced into an arteriotomy hole, fed distally into the ICA, and advanced a predetermined distance located 8 mm from the carotid bifurcation toward the MCA. After a 60-min period of focal cerebral ischemia, the filament was gently removed (onset of reperfusion). The collar suture at the base of the ECA stump was then tightened. The skin incision was closed and anesthesia was discontinued. Sham mice underwent neck dissection and coagulation of the ECA, but the MCA was not occluded. The success rate of the MCAO operation was approximately 90%. The animals were randomly divided into groups according to computer-generated randomization schedules, and blinding to group allocation was ensured. Dead animals and animals in which the MCAO model failed were excluded from the experiments. The animals were intraperitoneally injected with vinpocetine (10 mg/kg) after MCAO.[21, 25] The 10 mg/kg dose was selected for both compounds as optimal dose based on previous in vivo studies.

### Cell culture and OGD

As reported in previous studies, cortices were isolated from the embryonic day 16 (E16) wild type (WT) mice.[38] Primary cortical neurons were cultured in Neurobasal media (containing 4.5 g/l glucose, supplemented with GlutaMAX and B27; Life Technologies) for 7 d; microglia were cultured in 10% fetal bovine serum (FBS, Sigma-Aldrich) for 14 d. As described in our previous study [14], the oxygen and glucose deprivation (OGD) treatment was performed by removing the culture medium and replacing it with D-Hank's solution (Life Technologies); the cultures were then incubated in an anaerobic atmosphere of 95% N2 and 5% CO2 at 37°C for 3 h. OGD was terminated by replacing the D-Hank's solution with normal culture medium and returning the cells to a normoxic incubator. Prior to the OGD treatment, different concentrations (5, 20, or 50 μmol/L) of vinpocetine (Gedeon Richter Plc, China) were added to the culture medium. Control plates were maintained in the normoxic incubator during the OGD interval. Assays were conducted using 96-well microtiter plates. Cell viability was assessed with the CCK-8 assay, according to the manufacturer's protocols. Cell viability is expressed as a percentage of the control.

### Assessment of the cerebral infarct volume

The cerebral infarct volume was determined using a previously described method.[39] Forty-eight hours after the MCAO operation, the animals were sacrificed and perfused with ice-cold PBS via the ascending aorta. The brains were removed and sectioned coronally (25 μm). The slices were stained with a 2% triphenyl tetrazolium chloride (TTC) solution (Sigma-Aldrich, St. Louis, MO) for 15 min at 37°C. Normal brain tissue was stained brightly, whereas the infarcted areas were pale white. We measured the entire area of the prosencephalon and the cerebral infarct with Image-Pro Plus 5.0 image processing software (Media Cybernetics, Rockville, MD). The volumes of these areas were calculated using the formula V=t*(A1+A2+…An), where V is the volume of the infarct or prosencephalon, t is the thickness of the slice, and A is the infarct size and the volumetric ratio of the cerebral infarct (cerebral infarction volume/prosencephalon volume).

### Evaluation of neurological scores

Animals were scored using a previously described method[40]. Briefly, the mouse scoring system was: grade 0, no observable neurological deficits; grade 1, failed to extend the right forepaw; grade 2, circled to the right; grade 3, fell to the right; grade 4, could not walk spontaneously; and grade 5, dead. Scoring was performed by two trained investigators who were blinded to the animal group designation, and the mean score of the subscales was used as the final score for each animal.

### TUNEL and Fluoro-Jade B staining

TUNEL and Fluoro-Jade B (FJB) staining were performed according to the manufacturers’ protocols. The animals were deeply anesthetized and perfused with cold PBS followed by 4% paraformaldehyde through the ascending aorta, and the brains were sectioned coronally into 20-mm-thick frozen slices. TUNEL staining was performed using an In Situ Cell Death Detection kit (Roche, Indianapolis, IN). Cell degeneration was detected using an FJB staining kit (Millipore, Billerica, MA). Both assays were performed according to the manufacturers’ protocols. The stained sections were photographed with a confocal fluorescence microscope (Leica, Wetzlar, Germany). The nuclei were stained with DAPI^+^ (blue), and the apoptotic cells were TUNEL^+^ (green). We calculated the number of DAPI^+^ cells (blue) and TUNEL^+^ cells (green). Four quadrants were selected from each section, and the number of positive cells in each quadrant was counted. The average value was then calculated.

### Flow Cytometry

As described in a previous study[41], the medium was removed after the vinpocetine and OGD treatments and pooled with trypsinized adherent cells. Then, the trypsin was neutralized and the mixture was rotated at 1000 rpm for 5 minutes. Next, cells were washed and resuspended in cold PBS. The density of the cells was adjusted to 10^6^ cells per well by adding buffer. Next, 5 μL of PE and 5 μL of 7AAD were added, respectively, and incubated at room temperature in the dark for 30 min. Cells were filtered with a nylon mesh filter and subjected to flow cytometry.

### Western blotting

As described in our previous report[14], the mice were sacrificed 48 h after the induction of cerebral ischemia and the brains were removed. Following 3 h of OGD, the cells were collected. Proteins were resolved by sodium dodecyl sulfate–polyacrylamide gel electrophoresis (SDS-PAGE) and transferred onto polyvinylidene fluoride membranes by electroblotting. The membranes were incubated with 1:1000 dilutions of anti-TLR4 (Abcam, Cambridge, UK), anti-Myd88 (Abcam, Cambridge, UK), anti-TRIF (Abcam, Cambridge, UK), and anti-NF-кB p65 antibodies (Cell Signaling Technology, Danvers, MA, USA) overnight at 4°C. GAPDH (1:200; Santa Cruz Biotechnology, Dallas, TX, USA); β-actin (1:2000, Abcam, Cambridge, UK) and Lamin B (1:1000, Santa Cruz Biotechnology, Dallas, TX, USA) were used as loading controls. Membranes were subsequently incubated with a peroxidase-conjugated secondary antibody (Cell Signaling Technology, Danvers, MA, USA). The signals were detected using an ECL system, and the membranes were reprobed with an anti-GAPDH antibody (Santa Cruz Biotechnology). The signals were quantified by scanning densitometry using a bioimaging analysis system (LabWorks analysis software; UVP, Upland, CA).

### Real-Time Polymerase Chain Reaction

Real-time polymerase chain reaction (RT-PCR) was performed according to the manufacturer's instructions (Takara Biotechnology, Dalian, China). Ischemic brain tissue was rapidly harvested 24 h after cerebral I/R. Total RNA was extracted with TRIzol (Invitrogen, Gaithersburg, MD, USA) according to the manufacturer's instructions. The cDNA samples were synthesized using the iScript cDNA synthesis kit (Bio-Rad, Hercules, CA, USA) and real-time RT-PCR was performed on a Bio-Rad iCycler with the iQ SYBR Green Supermix (Bio-Rad) in 96-well plates. Primers were purchased from Shanghai Sangon Biological Engineering (Shanghai, China). The primer sequences were as follows: IL-1β, forward, gcccatcctctgtgactcat, reverse, agctcatatgggtccgacag; and TNF-α, forward, agaagttcccaaatggcctc, reverse, ccacttggtggtttgctacg. A threshold cycle value (CT) was calculated using the previously reported ΔΔC_T_ method [42] to quantify gene expression.

### Statistical analysis

All data are expressed as means±SEM. Differences between multiple groups were examined using one-way ANOVA and Bonferroni's post hoc test, and independent sample t tests were used for comparisons between two groups. When the data were not normally distributed, the nonparametric Kruskal-Wallis and Mann-Whitney *U* tests were used. A *p* value < 0.05 was considered statistically significant.
